# High current–density anodic electrodissolution in flow–injection systems for the determination of aluminium, copper and zinc in non–ferroalloys by flame atomic absorption spectrometry

**DOI:** 10.1155/S1463924699000036

**Published:** 1999

**Authors:** César Augusto Giacomozzi, Roldão R. U. de Queiróz, Ivan Gonçalves Souza, José Anchieta Gomes Neto

**Affiliations:** 1 Departamento de Química Universidade Federal de Santa Catarina PO Box 476 Florianópolis SC CEP 88040-900 Brazil; 2 Departamento de Química Analítica Instituto de Química UNESP PO Box 355 Araraquara SP CEP 14800-900 Brazil

## Abstract

An automatic procedure with a high current-density anodic electrodissolution unit (HDAE) is proposed for the determination of aluminium, copper and zinc in non-ferroalloys by flame atomic absorption spectrometry, based on the direct solid analysis. It consists of solenoid valve-based commutation in a flow-injection system for on-line sample electro-dissolution and calibration with one multi-element standard, an electrolytic cell equipped with two electrodes (a silver needle acts as cathode, and sample as anode), and an intelligent unit. The latter is assembled in a PC-compatible microcomputer for instrument control, and for data acquisition and processing. General management of the process is achieved by use of software written in Pascal. Electrolyte compositions, flow rates, commutation times, applied current and electrolysis time were investigated. A 0.5 mol l^-1^ HN0_3_ solution was elected as electrolyte and 300 A/cm^2^ as the continuous current pulse.

The performance of the proposed system was evaluated by analysing aluminium in Al-alloy samples, and copper/zinc in brass and bronze samples, respectively. The system handles about 50 samples per hour. Results are precise (R.S.D. < 2%) and in agreement with those obtained by ICP-AES and spectrophotometry at a 95% confidence level.


Journal of Automated Methods & Management in Chemistry, Vol. 21, No. 1 (January–February 1999) pp. 17–22

### High current-density anodic electro-dissolution in flow-injection systems for the determination of aluminium, copper and zinc in non-ferroalloys by flame atomic absorption spectrometry

**César Augusto Giacomozzi, Roldão R. U. Queiróz, Ivan Gonçalves Souza and José Anchieta Gomes Neto***

*Departamento de Química, Universidade Federal de Santa Catarina, PO Box 476; CEP 88040-900, Florianópolis-SC, Brazil, * Departamento de Química Analítica, Instituto de Química - UNESP, PO Box 355, CEP 14800-900, Araraquara-SP, Brazil*

*An automatic procedure with a high current-density anodic electro-dissolution unit (HDAE) is proposed for the determination of aluminium, copper and zinc in non-ferroalloys by flame atomic absorption spectrometry, based on the direct solid analysis. It consists of solenoid valve-based commutation in a flow-injection system for on-line sample electro-dissolution and calibration with one multi-element standard, an electrolytic cell equipped with two electrodes (a silver needle acts as cathode, and sample as anode), and an intelligent unit. The latter is assembled in a PC-compatible microcomputer for instrument control, and for data acquisition and processing. General management of the process is achieved by use of software written in Pascal. Electrolyte compositions, flow rates, commutation times, applied current and electrolysis time were investigated. A 0.5 mol $l^{-1}$ $HNO_3$ solution was elected as electrolyte and 300 A/$cm^2$ as the continuous current pulse.*

*The performance of the proposed system was evaluated by analysing aluminium in Al-alloy samples, and copper/zinc in brass and bronze samples, respectively. The system handles about 50 samples per hour. Results are precise (R.S.D. < 2%) and in agreement with those obtained by ICP-AES and spectrophotometry at a 95% confidence level.*

### Introduction

Aluminium and brass alloys are widely employed in automotive, aeronautic and railway industries, in the electric packaging section, civil and mechanical engineering, etc. due to the favourable physical characteristics of this metal, e.g. low-density, high resistance/weight ratio, high resistance to corrosion, high electric and thermal conductivity, suitability for superficial treatments, etc. [1–3].

According to a recent review made by Dulski [4], the analytical techniques usually employed in industrial quality control are optic emission spectroscopy (AOES), X-ray fluorescence spectroscopy (XRF), inductively-coupled plasma mass spectroscopy (CP-MS), inductively-coupled plasma atomic emission spectrometry (ICP-AES), atomic absorption spectrometry (AAS) and spectrophotometry. The first two tools are characterized by relatively specific and fast techniques, since the analysis is performed directly on the metal surface. The others, in spite of their good sensitivity, simplicity and low operational cost, determine elements only in liquid samples [5, 6].

Among the available techniques for direct metallic alloy analysis are spark and AC/DC arc [4, 7, 8], glow discharge (GDL) [4, 9] and laser ablation [4, 10, 11]. Although efficient, these accessories are relatively expensive and hardly portable. The anodic electro-dissolution approach (AE) [12–15] has been suggested as an alternative procedure for solid sample dissolution due to its simplicity, low operational cost, portability and feasibility of coupling with flow-injection analysis (FIA) [16]. On-line metallic sample dissolution, based on FIA-ED coupling, is very attractive in analytical routine work involving spectrometric or spectrophotometric determinations [17, 18].

In spite of the wide applicability of ED technique, the required solid standards for building analytical curves are the Achilles' tendon of this approach [13–15]. This cumbersome procedure can be circumvented by using an electro-dissolution cell which operates at close to 100% current efficiency. Although existing electro-dissolution cells with different configurations have been used for analytical routine work [12–15, 17, 19], an electrochemical cell operating at high current efficiency has not yet appeared in scientific literature.

This study reports on a new procedure for determining Al, Cu and Zn in non-ferroalloy samples by exploiting the approach of high current-density anodic electro-dissolution (HDAE) for on-line metallic sample dissolution in a flow-injection system.

### Experimental

### *Reagents, standards and samples*

All solutions were prepared with pro-analysis chemicals and distilled–deionized water (Milli-Q, Millipore).

The electrolyte was 0.5 mol $l^{-1}$ $HNO_3$ solution, prepared by diluting the concentrated acid.

Aluminium stock solution (100 mg $l^{-1}$) was prepared by dissolving 100 mg Al wire (Riedel, 99.999%) in 5 ml of hydrochloric acid. The solution was evaporated to near

1463-9246/99 $12·00 © 1999 Taylor & Francis Ltd dryness and the residue dissolved in 20 ml 0.25 mol $l^{-1}$ HCl. The volume was made up to 1000 ml with 0.25 mol $l^{-1}$ HCl.

Copper stock solution (100 mg $l^{-1}$) was prepared by dissolving 100 mg Cu powder (Aldrich, 99.99%) in 10 ml nitric acid and, after dissolution, the volume was made up to 1000 ml with water.

Zinc stock solution (100 mg $l^{-1}$) was prepared by dissolving 100 mg Zn powder (Aldrich, 99.99%) in 10 ml concentrated nitric acid. The resulting solution was diluted to 1000 ml with water.

Before the analysis, the metallic samples were treated by polishing their surfaces with sandpaper #400. After, they were washed thoroughly with water.

*The flow-injection system*

A Hitachi Model Z-8200 atomic absorption spectrometer, a home-made multi-channel peristaltic pump computer controlled equipped with Tygon® pumping tubes, polyethylene tubes (i.d. = 0.8 mm), Cole Parmer Model 98300-62 three-way solenoid valves, and connectors were used. The control and management of the process was performed by an IBM PC/AT-486 microcomputer by using a program written in Turbo Pascal. One power driver was based on the ULN 2004 and ULQ 2804 integrated circuits from SGS-THOMSON [20]. Microelectronics were employed in order to switch the continuous current source, peristaltic pump and solenoid valves. The microcomputer operated at a constant current source based on the LM350 integrated circuit from Texas Instruments [20], and this circuit is depicted in figure 1.

The source of current controlled by the personal computer (figure 1) was designed to have the capacity to supply up to 16 levels of constant current. The integrated

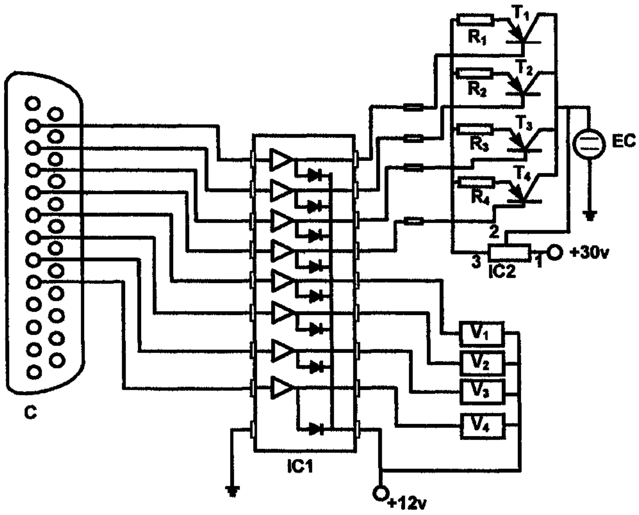

*Figure 1. Schematic diagram of the electronic circuit of the analyser. C: DB 25 centronics standard connector; $IC_1$: power driver (ULN2004A); $IC_2$: current regulator (LM350T), EC: electrolytic cell; $V_1$–$V_4$: solenoid valves; $R_1$–$R_4$: limiting current resistors; $T_1$–$T_4$: power transistors (TIP 32).*

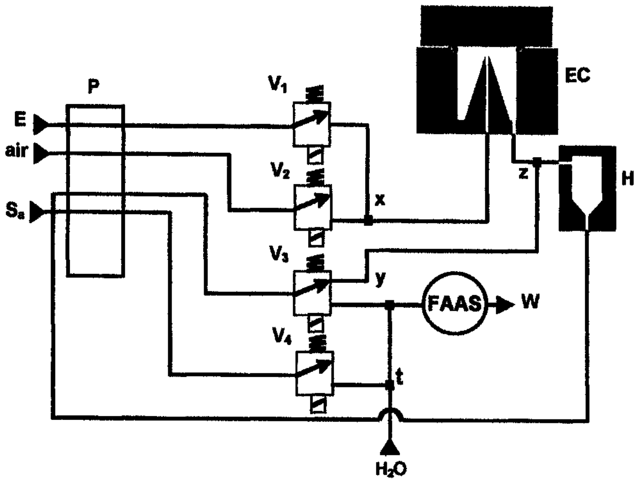

*Figure 2. Flow diagram. PP: Peristaltic pump; E: Electrolyte (0.5 mol $l^{-1}$ $HNO_3$); $S_a$: Analytical solution; $V_1$–$V_4$: solenoid valves; x, y, z, t: confluent points; EC: electrolytic cell; H: homogenization chamber; W: waste; FAAS: detector (flame atomic absorption spectrometer).*

circuit IC1 was used in the configuration of a power driver in order to provide an interface between the current source and the solenoid valves ($V_1$–$V_4$), and to the microcomputer. In the circuit of the current source, LM350T (IC2) was used, a positive voltage regulator of three terminals capable of supplying up to 3.0 A of maximum current. This device was used as a current precision regulator by connecting resistors $R_1$–$R_4$ between pins 1 and 2 of this component. Each resistor defines the level current supplied by the circuit. The binary combination of $2^4$ of bits 2–5 of the parallel port of the microcomputer (C) sets at a low level the exit pins of IC1 (1–4), saturating transistors $T_1$–$T_4$, which, switching the resistors with IC2, supplies the 16 current levels demanded by the process.

The electro-dissolution cell (EC, figure 2) used in this work was based on that proposed by Souza *et al.* [18]. It was machined in a perspex block acrylic resin. The cell compartment consisted of a 1 ml well, machined in a resin block.

A 2 mm hole in the bottom of the well was made in order to fix a silver needle (cathode). The electrolyte solution was injected into the cell through this electrode. After the electrolysis step, the inner solution was removed from the EC to the chamber H by a hole located on the lower right-side. A 1 mm hole in the top of the cell promoted the gas purge originated during the electrolysis. The circular site engraved on the upper surface of the chamber with a latex ring avoided the leakage of solution when a sample was positioned in the chamber.

The adjustable sample holder was used for entrapping samples of variable thickness on the electro-dissolution chamber. This procedure allowed accommodation of samples with a height of up to 5 cm. Electrical contact was made by means of this metallic holder.

### Procedure

The flow diagram of the system used for the determination of aluminium, copper and zinc in non-ferroalloys is depicted in figure 2. It consists of a solenoid valve-based commutation ($V_1$–$V_4$) in a flow-injection system for on-line sample electro-dissolution and calibration with one multi-element standard. After pre-treatment, the solid sample is positioned in the EC chamber and clamped by the adjustable holder. The start of an analytical cycle begins through a microcomputer command which activates the peristaltic pump (P) for approximately 30 s. This period is necessary for cleaning the transmission lines, removing the air bubbles and stabilizing the flow rates. Thereafter, the system calibration is carried out by opening the solenoid valve $V_4$ for various periods of time. In this situation, each multi-element standard volume undergoes a time-based injection into a carrier stream (water), and the established plug is directed towards the detector (FAAS). The passage of the processed aliquot through the spray chamber results in a large dispersion of the analyte. Transient atomic signals are measured as a peak with height proportional to the analyte content in the injected solutions. After maximum peak measurement, another cycle starts. With this procedure, only one 100 mg $l^{-1}$ standard solution containing Cu, Zn and Al (Sa) is sufficient for system calibration. The signal–time diagram of the process for on-line standard dilution by opening the valve $V_4$ is depicted in figure 3(*a*). When this valve opens, the pulses $P_1$–$P_5$ are at a low level. The times of pulses $P_6$ are fitted in a linearly crescent manner in order to obtain different aliquots of the standard solution to be injected into the system.

The signal–time diagram of an analytical cycle involving electrolysis is depicted in figure 3(*b*). The peristaltic pump is switched on again for 30 s by a $P_1$ pulse at high level. Two seconds after $P_1$, a pulse $P_2$ opens the solenoid valve $V_1$ for 7 s and electrolyte solution (E) is injected into the EC. Three seconds after $P_1$, another pulse $P_3$ turns the electro-dissolution source on for 5 s. The $P_3$ interval was longer than the electrolysis time in order to avoid the absence of electrolyte solution during electrolysis. After 7 s, $P_2$ and $P_3$ are at a low level again. The pulse $P_4$ is set at a high level and this opens the solenoid valve $V_2$ for 6 s. Air is directed towards EC and helps the electro-dissolved material removal from this chamber to the H chamber. This is achieved by aspirating the electro-dissolved material through the bottom of the H chamber and by opening the solenoid valve $V_3$. The solution recycles to the H chamber by a lateral orifice on the upper left-side of the chamber. After this operation, a pulse $P_5$ closes the $V_3$ valve for 2 s and the processed sample aliquot is injected into the carrier stream (water, 6 ml min$^{-1}$). This continuous-flow channel also permits efficient nebulizer aspiration when the $V_3$ valve is open.

A Thermo Jarrell Ash Model IRIS CID-DUO inductively coupled plasma atomic emission spectrometer and a Perking Elmer Model Lambda 11 spectrophotometer were used for the analysis of the samples and comparing the results as recommended.

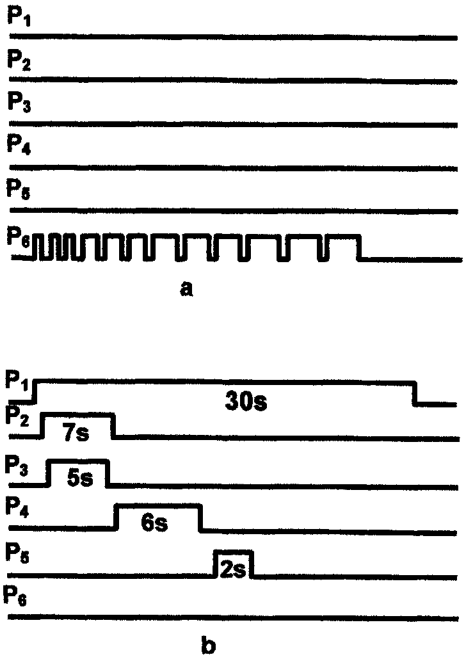

*Figure 3. Signal–time diagram of analyser for analytical curve generation (a) and analytical code (b). (a) Corresponds to various opening times of $V_4$ valve. A 100 mg $l^{-1}$ multi-element solution (Al, Cu and Zn) was used. (b) The pulses $P_1$, $P_2$, $P_3$, $P_4$ and $P_5$ correspond to peristaltic pump starting, $V_1$ opening time, electrolysis time, and $V_2$ and $V_3$ opening times, respectively.*

### Results and discussion

The flow diagram depicted in figure 2, and used for Al, Cu and Zn determinations in brass and Al-alloy samples, was designed as a compromise between system hydrodynamic pressure, electrolyte residence time and flow rate. Thermal oscillations markedly affected the conditions of direct current established at the beginning of the procedure because of the enormous dependence of electrolyte ionic conductivity on temperature [21]. The electrolyte, E, flowing through the cathode, is introduced into EC and reaches the sample (anode) in jet form. Besides dissipating the heat generated in the inter-electrodic region [21, 22], the higher rotation speed of the peristaltic pump promotes the continuous removal of the product of reactions from the anodic surface. The occurrence of secondary reactions is then drastically reduced [23]. During electrolysis, the concentration of the metallic ion produced in adjacencies of the anodic surface is very different from that produced in the bulk of the solution. The establishment of a concentration gradient implies the reduction of ionic mobility and conductivity of solution because the mass transport is governed by a pure diffusion mechanism [18]. Under conditions of high flow rate and low residence time of the electrolyte, the diffusion layer is minimized and the conductance is kept almost constant.

The influences of the current intensity and the electrolysis time on the amount of electrochemically removed material are presented in figures 4 and 5, respectively.

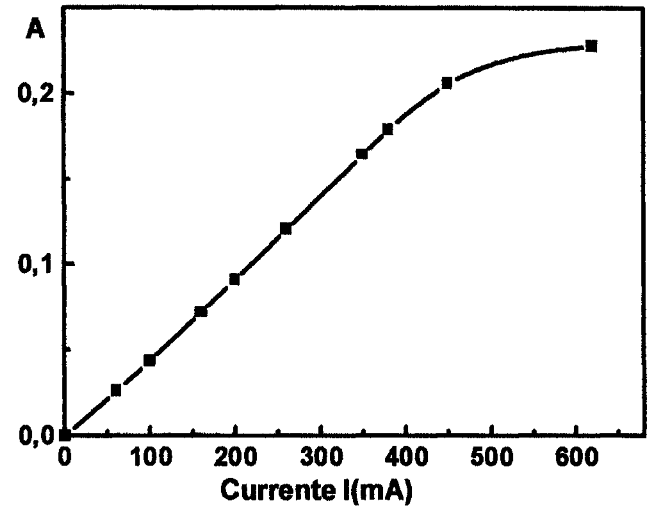

*Figure 4. Influence of applied current on signal. The figure refers to the proposed system with a 3s electrolysis time; the curve corresponds to Al-sheet A2. A = integrated absorbance.*

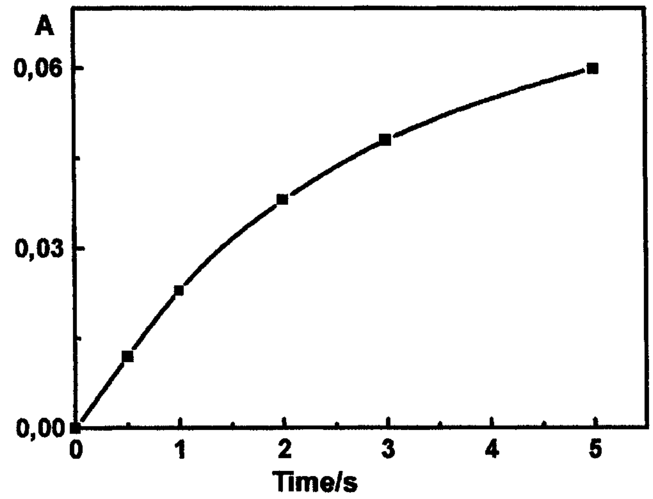

*Figure 5. Influence of electrolysis time on signal. The figure refers to the proposed system with a 250mA current; the curve corresponds to Al-sheet A2. A = integrated absorbance.*

In both cases, the efficiency of the process increased with either current increase or electrolysis time. This effect was based on the Faraday law [24] which indicates that the quantity of the electro-dissolved mass depends directly on the Coulomb quantity. Aiming to maintain a compromise between analytical rate and low current, the subsequent studies were carried out with 250 mA current and 3 s electrolysis time.

With regard to selected Al-alloy samples, iron, copper, magnesium, lead, tin and silicon are the majority constituents. Alloys having very different electrode potentials present preferential dissolution of the metal with smaller reduction potential. This is very critical for low-current density conditions. On the other hand, high-density current conditions minimize this effect, since the ddp between anode and cathode is sufficiently high to guarantee adequate potentials, making the dissolution of the constituents easily attainable. As the current density and inter-electrodic distance are inversely proportional, higher efficiency in the electro-dissolution process was observed when the inter-electrodic distance was maintained at 500 μm. This permitted the performing of the electrolysis under high-density current conditions. The electrochemical cell configuration adopted in this work has been efficiently applied to the routine analysis of nonferrous alloys since current densities up to 600 $A/cm^2$ are easily attained. In this way, the on-line anodic electro-dissolution proposed by Bergamin *et al.* [15] was improved in electro-dissolution systems involving high-efficiency currents, as suggested by Souza *et al.* [18].

In the passive region of potential, aluminium is one metal which is characterized by the formation of self-protecting films with extremely low conductivity [22]. At high anodic potentials, the thickness of the film can increase significantly. Indeed, aluminium anodization depends on the electrolyte composition. This can originate film of several μm thickness which can stand a potential difference higher than 100 V [22]. The aluminium dissolution which uses high-density current (transpassive region) involves localized rupture of the passive film when concentrated acids are involved [22]. In this sense, the electrolyte composition is a relevant parameter in this procedure. Strong inorganic acid solutions were elected as electrolytes because they avoid the formation of insoluble products. The presence of this anodic mud in the flow-system may result in line-clogging. The influence of the nature of the electrolyte on the analytical signal is depicted in figure 6.

Sulphuric acid solution was the worst electrolyte. This is probably due to its characteristic passivity. Although the signals obtained with hydrochloric acid-based electrolytes were higher than those obtained with sulphuric acid, these solutions also presented passivity properties. Even at high concentration and current density, chloride ions generated a thin layer on the electrolysed sample surface. When the electrolyte was a nitric acid solution,

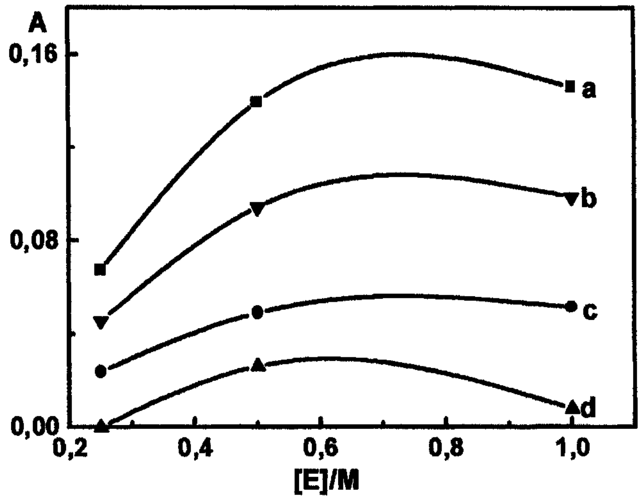

*Figure 6. Influence of electrolyte nature on signal. The figure refers to the Al-sheet A2 analysed with the proposed system at 250 mA current and a 3 s electrolysis time. (a)–(d) Correspond to $HNO_3$, $HNO_3$ plus HCl, HCl and $H_2SO_4$, respectively. A = integrated absorbance.*

the analytical signals increased by about 50% for Al, Cu and Zn, thus improving the sensitivity and linearity of the calibration curves. Since good results were obtained for 0.5 mol $l^{-1}$ $HNO_3$ solution, this was the electrolyte concentration selected for this work. It should be stressed that this is in agreement with studies dealing with non-ferrous alloys [25].

The system calibration in direct solid analysis based on the electro-dissolution technique can be carried out by using aqueous standards instead of solid ones. The main requisite is an electrochemical cell operating at high anodic-current density in order to permit sample dissolution at high removal rate. In this way, the calibration of the system with aqueous standards was elected. However, the conventional manual preparation of standard solution sets is laborious and time-consuming, so an auto-diluter system was adopted as an alternative analytical tool. This calibration procedure combined the use of FIA and pneumatic nebulizer to dilute on-line a standard solution, as recommended by Gomes Neto *et al.* [26]. With this procedure, different aliquots of only one analytical solution (Sa) were sequentially injected into the nebulizer stream (water) by opening valve $V_4$ for various periods of time. Transient atomic signals recorded as a peak with area proportional to the injected volume, were obtained. Analytical curves for copper, aluminium and zinc were then easily and efficiently constructed. The main parameters related to analytical curves built were flow rates, standard solution concentration and commutation time of the $V_4$ valve. With the flow rate and concentration of Sa at 2.5 ml $min^{-1}$ and 100 mg $l^{-1}$, respectively, a good correlating coefficient (0.9999) between integrated absorbance and injection time in the 250–6000 ms range was obtained for Al, Cu and Zn. The influence of $V_4$ valve timing on the signal of a 100 mg Al $l^{-1}$ solution is depicted in figure 7.

With regard to the quantification of the analytes in the solid samples, it should be commented that the analytical signal of any electrolysed sample volume injected into the AAS should be within the absorbance values corresponding to 250 and 6000 ms $V_4$ opening time. As the opening time for the introduction of a processed sample volume is variable, the following protocol is used for the analyte quantification: for a given $V_3$ valve opening time ($t_s$; sample injection time) that produces a signal $S_1$ ($S_1$ should be fitted in the linear calibration range), select the $V_4$ valve opening time on the calibration curve ($t_{std}$, standard injection time) that coincides with the $S_1$ value; thereafter, by applying the law of proportions, the analyte concentration ($C_a$) can be computed by $C_a = (t_{std}/t_s)C_{std}$.

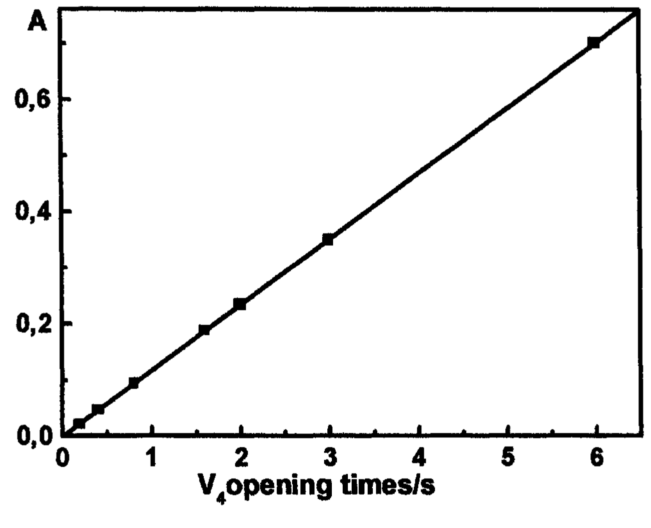

*Figure 7. Influence of $V_4$ valve opening time on signal. The figure refers to the auto-diluter system with a 100 mg Al $l^{-1}$ solution. A = integrated absorbance.*

The spectrometer readings were transferred to the microcomputer through a serial port. During data acquisition, the signals from both the analytical solution aliquots and the samples were stored in an ASCII file. After acquisition, the software computed the corresponding peak area, plotted the analytical curves and estimated the analyte concentration.

With the proposed system, aluminium was determined in several Al-alloy samples (table 1), and copper and zinc were determined in brass samples (table 2).

Results were precise (R.S.D. < 2%) and in agreement with those obtained by ICP-AES and spectrophotometry at a 95% confidence level. With this system, 50 samples could be run per hour. The on-line electrochemical dissolution with an auto-diluter system improved the sampling rate and quality of the analytical results by

*Table 1. Comparative results. Zinc and copper contents in brass (in %) as determined by the system of figure 2 (FIA) and by inductively-coupled plasma atomic emission spectrometry (ICP-AES). Numbers in brackets (in %) are estimates of R.S.D. based on three replicates.*

| | *Zn* | | *Cu* | |
|---|---|---|---|---|
| *Sample* | *ICP-AES* | *FIA* | *ICP-AES* | *FIA* |
| 1 | 6.04 (6.2) | 6.50 (1.8) | 76.87 (2.3) | 78.00 (0.9) |
| 2 | 34.94 (3.2) | 37.03 (0.7) | 67.92 (1.7) | 68.67 (1.0) |
| 3 | 36.22 (1.6) | 34.16 (0.9) | 61.76 (1.3) | 60.01 (0.8) |
| 4 | 37.77 (2.0) | 38.90 (0.6) | 62.12 (2.1) | 63.05 (0.8) |
| 5 | 35.82 (0.6) | 36.50 (0.6) | 61.68 (3.4) | 62.89 (0.7) |
| 6 | 36.22 (1.7) | 35.00 (1.0) | 61.25 (2.5) | 63.10 (0.8) |
| 7 | 36.10 (2.8) | 36.70 (0.8) | 60.81 (4.2) | 61.53 (1.2) |
| 8 | 36.92 (2.5) | 35.00 (1.2) | 61.76 (3.7) | 60.35 (1.1) |

*Table 2. Comparative results. Aluminium contents in Al standard (Riedel) and Al-sheets (in %) as determined by the proposed procedure (FIA) and by spectrophotometry (UV-VIS). Numbers in brackets (in %) are estimates of R.S.D. based on three replicates.*

| *Sample* | *UV-VIS* | *FIA* |
|---|---|---|
| Riedel | 98.14 (4.1) | 98.25 (2.3) |
| A1 | 93.15 (2.6) | 93.18 (3.2) |
| A2 | 95.15 (3.2) | 95.25 (1.6) |
| A3 | 94.28 (5.0) | 94.47 (1.2) |
| A4 | 92.37 (3.7) | 92.73 (0.9) |

reducing the potential source of contamination which is present in conventional procedures.

### Conclusions

With the HDAE approach, the aqueous standard could be used for calibration. HDAE-FIA-FAAS coupling can be efficiently applied to aluminium, copper and zinc determination in Al-alloy and brass samples in large-scale analysis.

### Acknowledgments

The authors thank FUNDUNESP (Project 77/97) for the financial support of this work. CNPq and CAPES are thanked for the fellowships of J.A.G.N. and J.B.B.S., respectively.

### References

1. Weingaerter, W. L., 1990, *Tecnologia de usinagem do alumínio e suas ligas* (São Paulo: Alcan Alumínio do Brasil).

2. Coutinho, T. A., 1980, *Análise e prática: metalografia de não ferrosos* (Rio de Janeiro: Edgard Blücher), p. 42.

3. Long, F. J., 1965, Non ferrous metals and their alloys. *Student Aids to Engineering Workshop Technology* **II** (Moscow), pp. 52–90.

4. Dulski, T. R., 1993, *Analytical Chemistry*, **65**, 29.

5. Barnes, R. M., Lind, F., Jing, L. S. and Mahant, H. S. 1983, *Applied Spectroscopy*, **37**, 389.

6. Broekaert, J. A. C. and Boumans, P. W. J. M., 1987, in *Inductively Coupled Plasma Emission Spectrometry, Part I: Sample Introduction Techniques* (New York: John Wiley), pp. 296–395.

7. Ohls, K. and Sommer D., 1979, *Fresenius, Journal of Analytical Chemistry*, **296**, 241.

8. Farnsworth, P. B. and Hifftje, G. M., 1983, *Analytical Chemistry*, **55**, 1414.

9. Caroli, S., 1983, *Spectrochimica Acta*, **47B**, 625.

10. Carr, J. W. and Horlich, G., 1982, *Spectrochimica Acta*, **37b**, 1.

11. Thompson, M., Ggouter, J. E. and Sieper, F., 1981, *Analyst*, **106**, 32.

12. Barrabas, S. and Lea, S. G., 1965, *Analytical Chemistry*, **32**, 1132.

13. Coutinho, C. A., Arruda, E. C. and Etrusco, G. S. P., 1983, *Metalurgia ABM*, **39**, 87.

14. Coutinho, C. A., Arruda, E. C. and Azeved, J. C., 1981, *Metalurgia ABM*, **37**, 683.

15. Bergamin, F. H., Krug, F. J., Zagatto, E. A. G., Arruda, E. C. and Coutinho, C. A., 1986, *Analytica Chimica Acta*, **109**, 177.

16. Ruzicka, J. and Hansen, E. H., 1975, *Analytica Chimica Acta*, **78**, 145.

17. Bergamin, F. H., Krug, F. J., Nobrega, J. A., Oliveira, P. V., Reis, B. F., Souza, I. G. and Mesquita, M., 1991, *Analytica Chimica Acta*, **214**, 397.

18. Souza, I. G., Bergamin, F. H., Krug, F. J., Nobrega, J. A., Oliveira, P. V. and Reis, B. F., 1991, *Analytica Chimica Acta*, **245**, 211.

19. Chirside, R. C., Cluley, H. J. and Proffit, P. M. C., 1957, *Analyst*, **82**, 18.

20. Motorola Analog IC Device Data, 1986, Motorola, **1**, 3.105.

21. Datta, M. and Landolt, D., 1983, *Journal of Applied Electrochemistry*, **13**, 785.

22. John, F. and Cahles, 1967, *Metals Handbook*, 8th ed., Vol. 3, Electrochemical Machine (ECM) (USA: Machining American Society for Metals), p. 233.

23. McGeough, J. A., 1974, *Principles of Electrochemical Machining* (London: Chapman and Hall).

24. Yuan, D. X., Wang, X. R., Yang, P. Y. and Huang, B. L., 1991, *Analytica Chimica Acta*, **243**, 65.

25. Hill, U. T., 1956, *Analytical Chemistry*, **28**, 1419.

